# Contrasting ecological niches lead to great postzygotic ecological isolation: a case of hybridization between carnivorous and herbivorous cyprinid fishes

**DOI:** 10.1186/s12983-021-00401-4

**Published:** 2021-04-21

**Authors:** Haoran Gu, Yuanfu Wang, Haoyu Wang, You He, Sihong Deng, Xingheng He, Yi Wu, Kaiyan Xing, Xue Gao, Xuefu He, Zhijian Wang

**Affiliations:** 1 Key Laboratory of Freshwater Fish Reproduction and Development (Ministry of Education), Key Laboratory of Aquatic Science of Chongqing, School of Life Sciences, Southwest University, Chongqing, 400715 China; 2 Shanghai Synchrotron Radiation Facility, Shanghai Advanced Research Institute, Chinese Academy of Sciences, Shanghai, 201204 China; 3 Liangshan Kehua Water Ecology Company Limited, Xichang, 615000 China; 4 Sichuan Lubei Biotechnology Company Limited, Chengdu, 610011 China; 5 Xichang Agriculture and Rural Affairs Bureau, Xichang, 615000 China

**Keywords:** Hybrid fitness, Foraging behavior, Feeding habit, Ecological niche, Morphology

## Abstract

**Background:**

Postzygote isolation is an important part of species isolation, especially for fish, and it can be divided into two aspects: genetic isolation and ecological isolation. With the increase in parental genetic distance, the intensity of genetic isolation between them also increases. Will the increase in parental ecological niche differences also lead to the increase in ecological isolation intensity between them? This question is difficult to answer based on the current literature due to the lack of hybridization cases of contrasting ecological niche parents.

**Results:**

Cyprinid fish parents (*Schizothorax wangchiachii* and *Percocypris pingi)* with contrasting ecological niches (herbivorous and carnivorous) and their F1 hybrids were used as research objects. Fish and periphytic algae were selected as food corresponding to different parental resources. The foraging-related traits of these hybrids are generally the same between parents; however, the intermediate foraging traits of hybrids did not result in intermediate foraging performance for parental resources, and these hybrids could hardly forage for parental resources. The poor foraging performance of these hybrids for parental resources was caused not only by the decline in the foraging ability of these hybrids but, more importantly, by the decrease in foraging activity. Interestingly, these hybrids initially showed a high interest in foraging small fishes; however, after the first successful capture, these hybrids had difficulty ingesting fish and spit them out, which led to the subsequent decrease in foraging activity. We designed a series of experiments to explore the mechanism of the fish spitting of these hybrids, excluding the taste and the size of prey, and found that the decrease in their pharyngeal tooth puncture ability may be the reason.

**Conclusions:**

This study was the first to demonstrate that these parents with contrasting ecological niches will produce great postzygotic ecological isolation for parental resources. The poor foraging performance of these hybrids for parental resources is mainly due to the decrease in foraging activity. Interestingly, these hybrids have obvious fish-spitting behaviour, which is a typical example of the incompatibility between intermediate traits and genetic behaviors.

**Supplementary Information:**

The online version contains supplementary material available at 10.1186/s12983-021-00401-4.

## Introduction

What are species? The biological species concept defines “species” as populations that can mate with each other and have isolating barriers with other populations, where isolation barriers can be divided into prezygote barriers and postzygote barriers [1]. Many advances have been made in the evaluation of ecologically associated prezygote barriers [2]. Prezygote barriers are often clearly associated with ecological divergence and contribute to isolating barriers, for example, via habitat isolation [3, 4], temporal isolation [5], sexual isolation [6], and mechanical isolation [7]. Postzygote barriers can be divided into two aspects [1]. First, intrinsic or genetic isolation reflects low hybrid fitness due to general genetic incompatibilities between the genomes of divergent populations [8, 9]. Second, extrinsic or ecologically dependent isolation specifically refers to reduced hybrid fitness due to the maladaptive intermediacy of their ecologically relevant genotypes and phenotypes in parental environments [10]. Due to the high number of species compared to other vertebrate taxa, coupled with in vitro fertilization, more cases of hybridization are observed in fish than other vertebrate clades [11–17]. Thus, the postzygote isolation is particularly important for fishes. Many studies have demonstrated intrinsic isolation, but the potential ecological contributions to postzygote isolation are also very important [18–21].

Regarding ecologically dependent isolation, important examples include an investigation of hybrids between the benthic and the limnetic forms of three-spine stickleback [10, 21]. Both F1 and F2 hybrids grew more poorly in the parental environments than each parent. There are many other similar examples found in leaf beetles, aphids, cichlid fishes, sunfishes, and so on [18, 22–25]. Why are such hybrids at a disadvantage in the parental environments compared to the parent species? Currently, the main explanation is the incompatibility between intermediate morphology [10, 21] or kinematics [25] of hybrids and the parental food resources.

**Fig. 1:**
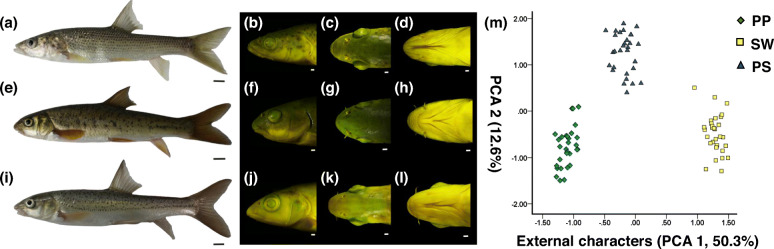
External characters comparison. **a** The full view of PP. **b**-**d** The head characters of PP. The full view of SW. **f**-**h** The head characters of SW. **i** The full view of PS. (j-i) The head characters of SP. (m) The PCA of external characters. The white scale is 1 mm; the black scale is 10 mm. We have used some figures in previous articles [27], including (**a**), (**c**), (**d**), (**e**), (**g**), (**h**), (**i**), (**k**) and (**l**)


Foraging process of a species is not only determined by foraging related traits, but also by the corresponding foraging behaviors, with a significant correlation between them [26]. For example, carnivorous fish have larger mouths and aggressive behavior, while herbivorous fish have smaller mouths and scraping behavior (Fig. 1 and Additional Movies 1–2). Foraging traits are often quantitative, and are therefore frequently additive between parents in F1 hybrids [21]. However, many unique parental genetic behaviours of F1 hybrids may be codominant [28] or dominant [29] rather than additive. These indicate that there may be incompatibilities between asymmetric traits and behaviours in F1 hybrids.

**Additional file 1: Movie 1.**


**Additional file 2: Movie 2.**

Regardless of the presence of prezygote barriers or postzygote barriers, interspecific isolation intensity typically increases with the increase in genetic distance between populations, and the postzygote genetic isolation intensity can be measured by the survival and fertility of hybrids [19, 30]. Therefore, will the increase in ecological niche differences also lead to the increase in ecological isolation intensity? The current research cannot easily answer this question due to the lack of hybridization cases of contrasting ecological niche parents. Fortunately, we have recently obtained healthy, morphologically and genetically stable F1 hybrids (PS) crosses of carnivorous and herbivorous cyprinid fishes (Fig. 1) [27]. Both parents used in this study were cold-water Cyprinidae fishes from the upper Yangtze River basin in the south-eastern Tibetan Plateau, and they have similar breeding periods. *Schizothorax wangchiachii* (SW) has a sharp horny front jaw and mainly scrapes and eats periphytic algae from rocks (Fig. 1 and Additional Movie 1). *Percocypris pingi* (PP) is a typical carnivorous fish with a sub-superior mouth (Fig. 1 and Additional Movie 2). Morphologically, the *Schizothorax* genus and *Percocypris* genus were once thought to belong to two different subfamilies [31]. However, molecularly, they were shown to be sister genera in recent studies [32, 33]. Although they are sympatric and have similar breeding seasons, no hybrids have been reported in the wild, indicating strong isolating barriers between them.

To compare the ecological context dependency of interspecific hybridization we experimentally test the foraging ability and behavior of F1 hybrids and parental species for parental resources. This results in two main predictions:

1. Great ecological isolation exists between contrasting ecological niche parents, and F1 hybrids could hardly forage for parental resources.
2. There may be incompatibilities between foraging traits and foraging behaviors of F1 hybrids, which may have adverse effects.

To answer the above question, carnivorous fish, herbivorous fish and their F1 hybrids were used to explore the ecological adaptability of the F1 hybrids through comparative behavioural and morphological studies.

## Materials and methods

### Experimental fish acquisition

In March 2017 and 2019, a hybridization experiment and parental reproduction were performed; details on the methods can be found in the literature [27]. Age-two fishes (PP (122.03 ± 1.78 mm, 25.2 ± 1.05 g), SW (106.78 ± 1.41 mm, 18.43 ± 0.74 g) and PS (125.84 ± 2.71 mm, 29.22 ± 1.85 g)) were used to quantify both external and skeletal characteristics, and age-one fishes (PP (9.08 ± 0.34 mm, 12.07 ± 0.90 g), SW (9.23 ± 0.14 mm, 13.03 ± 1.50 g) and PS (9.17 ± 0.48 mm, 14.02 ± 3.76 g)) were used to quantify foraging and behavioural features.

### Food types

We used two types of food corresponding to parent resources. The first type consisted of small fishes, *Sinibrama taeniatus* (0.0507 ± 0.0043 g) or *Carassius auratus* (0.0748 ± 0.0023 g), the former of which is mainly distributed in the upper Yangtze River and the latter of which is widely distributed. The second type consisted of periphytic algae (*Spirogyra*, tough population on the pool wall or tender population on stone. Unfortunately, we did not produce the most palatable diatoms for SW in the pond; however, *Schizothorax* fishes, such as SW, still eat a certain amount of *Spirogyra* algae under natural conditions [34]), it is widely distributed and abundant in China’s water system.

### Morphology

The external morphology of age-two SW (*n* = 30), PP (*n* = 30) and PS (*n* = 30) was studied, and the examination standards are shown in Additional Table 2. Then, we random selected 10 fish individuals from each species for quantification of skeletal morphology. Their opercular bone, pharyngeal bone, dentary bone and skull were obtained by boiling, and the examination standards are described in Additional Fig. 1. Finally, 19 external morphological indicators and 19 skeletal morphological indicators were quantified in this study, as shown in Additional Tables 2–3. To visually show the comprehensive morphological differences between the three fishes, we conducted principal component analysis (PCA) of two categories of indicators (Additional Tables 4–7).

**Fig. 2:**
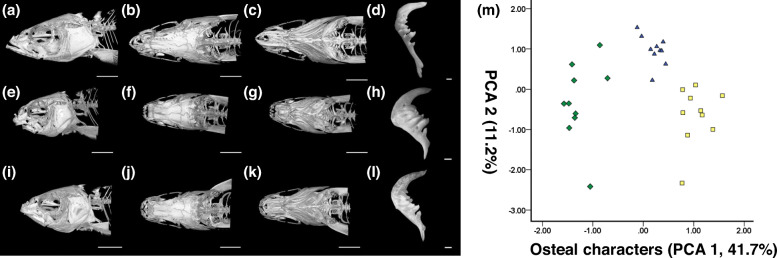
Osteal characters comparison. **a**-**c** The MicroCT image of head characters of PP. **d** The pharyngeal bone of PP. **e**-**g** The MicroCT image of head characters of SW. **h** The pharyngeal bone of SW. **i**-**k** The MicroCT image of side head of PS. **l** The pharyngeal bone of SP. **m** The PCA of osteal characters. The scale of MicroCT images is 6 mm, and the scale of pharyngeal bones is 1 mm

The body shapes were photographed using an SLR camera (Canon EOS 100D, Japan). The details of the heads fixed by Bouin’s fixative and bones were photographed (Fig. 1) by a stereomicroscope (Nikon SMZ25). Age-two PP, SW and PS were scanned (Fig. 2) using a MicroCT Skyscan 1176 (Bruker, Belgium) to obtain the holistic bone structure; specific methods are described in [35], and they were slightly modified in this study.

### Comparison of foraging habit

**Fig. 3:**
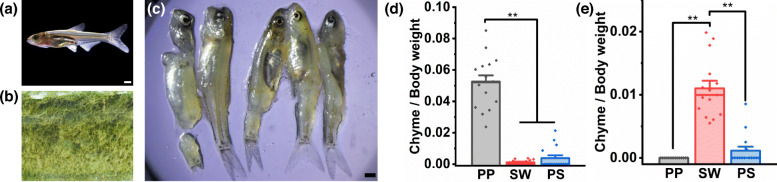
Comparison of foraging habit. **a** Small fish (*S. taeniatus*) **b** Tough periphytic algae (*Spirogyra*). (**c**) Small fish debris. **d** The FL (foraging level) of small fishes among PP, SW and PS. **e** The FL (foraging level) of tough periphytic algae among PP, SW and PS. The scale of all figures is 1 mm. The different ** above the boxes differ significantly at *P* < 0.01 based on Tukey test, the height give the mean, the thick lines give the medians, and whiskers indicate mean ± SE

We fed PP, SW and PS with small fishes (*S. taeniatus*) and tough periphytic algae on the pool wall (Fig. 3). After the experimental fish had adapted to the food for a period of time, we dissected them and weighed their chyme. Specific experimental methods can be found in Additional method 1. We compared each fish species’ foraging level (FL) using the following formula:

$$ FL=M2/\left(M1-M2\right) $$

where *M*1 represents body weight, and *M*2 represents chyme weight.

Due to the large number of quantitative indicators in this study, such as FL, the contents and abbreviations of all the quantitative indicators are shown in Additional Table 1 for the convenience of readers.

### Hybrid vs *P. pingi* in foraging fish

**Fig. 4:**
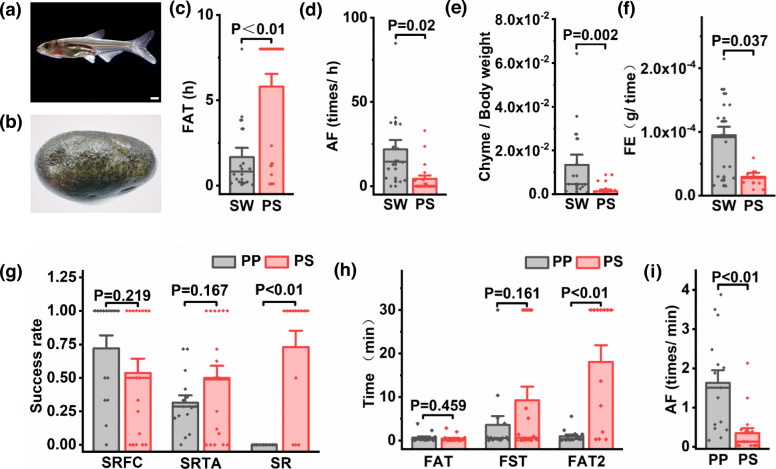
Hybrid vs parents in foraging little fish or periphytic algae. **a** Small fish (*S. taeniatus*). **b** Rock with tender periphytic algae (*Spirogyra*). **c** PS vs SW in the FAT (first attack time). **d** PS vs SW in the AF (attack frequency). **e** PS vs SW in the foraging level. **f** PS vs SW in the FE (foraging efficiency). **g** PS vs PP in the SRFC (success rate of the first successful capture), SRTA (success rate of the total attacks) and SR (spitting rate). **h** PS vs PP in the FAT, FST (first success time) and FAT2 (first attack time after the first successful capture). **i** PS vs PP in the AF (attack frequency). The scale is 1 mm. The numbers above the columns give the *P*-value based on Tukey test, the height give the mean, the thick lines give the medians and whiskers indicate mean ± SE

We compared the foraging capacity of PP (*n* = 15) and PS (*n* = 18) for small fishes (*S. taeniatus*) (Fig. 4a). Specific experimental methods are described in Additional method 2. We observed experimental fishes by video and quickly replayed the video and counted the following indicators: first attack time (FAT), first success time (FST), the success rate of the first successful capture (SRFC), first attack time after the first successful capture (FAT2), attack frequency (AF), the success rate of the total attacks (SRTA), and the spitting rate (SR). Details of these indicators are as follows:

FAT: The time when an experimental fish first attacked the small fishes. To exclude the influence of irritability, only the experimental fishes that launched the first attack within 5 min were included in all statistical comparisons.

FST: The time when an experimental fish first successfully caught a small fish. If it did not succeed within 30 min, a value of 30 min was used as its first success time.

SRFC: The success rate when an experimental fish first successful capture.

FAT2: The time when an experimental fish first attacked after the first successful capture.

AF: The average number of attacks per minute of an experimental fish; this value was calculated using the following formula:

$$ AF=N/T $$

SRTA: This value was calculated using the following formula:

$$ SRTA=N^{\prime }/N $$

SR: Some individuals catch fish and then spit them out; this value was calculated using the following formula:

$$ SR=N^{\prime\prime }/N^{\prime } $$

where *N* represents the total number of attacks; *T* represents the time at the end of the experiment; *N*′ represents the total catch before the end of the experiment (not intake); and *N* ′ ′ represents the number of fish spitted.

We compared the abilities of SW (*n* = 16) and PS (*n* = 20) to forage tender periphytic algae (Fig. 4b). Specific experimental methods are described in Additional method 3. We quickly replayed the video and evaluated the following indicators: FAT, AF, FL and foraging efficiency (FE). The details of these indicators are as follows

FAT: The time when an experimental fish first scraped periphytic algae from the rocks.

AF: The average number of scrapings per hour of experimental fish; this value was calculated using the following formula:

$$ AF=\left(N2+N5+N8\right)/3 $$

FE: The average weight of a single scrape of periphytic algae per unit weight of experimental fish; this value was calculated using the following formula:

$$ EF=M2/\left( AF\times 8\times \left(M1-M2\right)\right) $$

where *N*2, *N*5, and *N*8 represent the number of attacks in the second, fifth and eighth hours, respectively, *M*1 represents the body weight of the experimental fish; and *M*2 represents the chyme weight of the experimental fish.

### Assessment of whether the behaviour of hybrid fish spitting fish is persistent

**Fig. 5:**
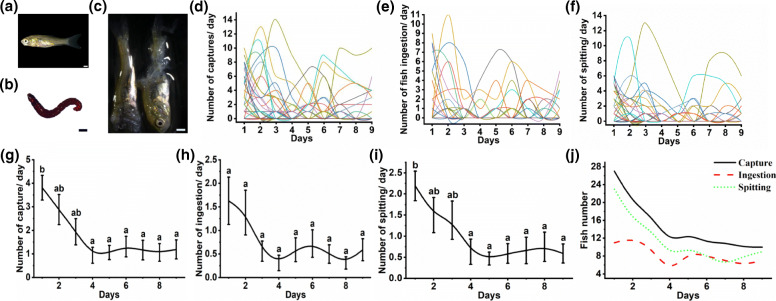
The changes of the related indicators of foraging fish in hybrid fish with time. **a** Small fish (*C. auratus*). **b** Blood worms (Chironomidae larvae). **c** Small fish debris. **d** The trends of captures of every SP with time. **e** The trends of ingestion of every SP with time. **f** The trends of spitting of every SP with time. **g** The mean trend of captures of SP with time. **h** The mean trend of ingestion of SP with time. **i** The mean trend of spitting of SP with time. **j** The trends in the number of SP involved in capture, ingestion and spitting. In **d**, **e** and **f**, each line represents an individual. The scale of all figures is 1 mm. The different superscripts **a**, **b** above the lines differ significantly at *P* < 0.05 based on Tukey test, and whiskers indicate mean ± SE


In the previous experiments, we observed that PS had obvious behaviour of spitting fish (Fig. 3c and Additional Movie 3). To test if this behavior is persistent, we set up a feeding experiment using small fish (*C. auratus* (Fig. 5a)) for 9 days, and PS still had obvious spiting behaviour after catching the small *C. auratus* fishes (Fig. 5c). For 9 days, we fed not only fish but also blood worms (Fig. 5b, 0.0171 ± 0.0006 g, Chironomidae larvae, a soft-bodied aquatic insect) to simulate a palatable food shortage, but not a complete absence, in the natural environment. Specific experimental methods are described in Additional method 4. We counted the daily catch, intake, and spitting of each PS for small fish.

**Additional file 3: Movie 3.**

### Mechanism explaining why hybrid fish spitted fish

**Fig. 6:**
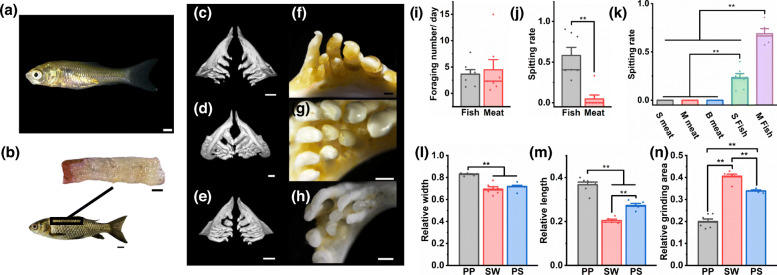
Mechanism of hybrid fish spitting small fish. **a** Small fish (*C. auratus*). **b** A small piece muscle in the back of *C. auratus*. **c**-**e** The MicroCT image of pharyngeal bones of PP, SW and PS. **f**-**h** The detail image of grinding surface of pharyngeal bones of PP, SW and PS. **i** The average number of daily foraging (ANDF) for small fish or meat by these SP with a persistent spitting-fish behavior. **j** Compare the spitting rate (SR) of SP between foraging small fish and meat. **k** Compare the SR of SP between foraging S fish (small fish, 0.09 ± 0.01 g), M fish (medium fish, 0.26 ± 0.03 g), S meat (small meat, 0.10 ± 0.01 g), M meat (medium meat, 0.24 ± 0.01 g), B meat (big meat, 0.50 ± 0.05 g). **l** The relative maximum opening width between pharyngeal teeth (MOWPT). **m** The development degree of hook pharyngeal teeth (DDHPT). **n** The relative grinding surface area of pharyngeal teeth (GSAPH). The scale in **a** is 1 mm, in (**b**), the meat is 1 mm and the fish is 10 mm, in (**c**-**e**) is 0.5 mm, in (**f**-**h**) is 2 mm. The different ** above the boxes differ significantly at *P* < 0.01 based on Tukey test, the height give the mean, the thick lines give the medians, and whiskers indicate mean ± SE

Two mechanisms may explain why PS spitted small fish: the small fish tasted bad or they were difficult to chew. To explore this mechanism, we selected approximately 50 g of *C. carp* (Fig. 6a) and cut the back muscle into small pieces (Fig. 6b) without bone, instead of using small fish. We took PS that had the obvious behaviour of spitting small fish in the last experiment as the experimental fishes (*n* = 7). Other than the small fishes that were replaced with small pieces of *C. carp* muscle, the other feeding and statistical schemes were the same as those in Section 2.7. However, the experiment lasted only 3 days. We counted the average number of daily foraging (ANDF) and the SR of the 7 experimental fishes used in Section 2.7 and this experiment, which was equivalent to the former serving as a control group for the latter, by the following formulas:

$$ ANDF=N/T $$

where *N* represents the total number of prey captured by PS during the experiment, and *T* represents the number of days of the experiment.

Next, to investigate whether prey size also leads to fish-spitting behaviour in PS, the SR of PS to different sizes of meat and fish was quantified. The specific experimental methods are described in Additional method 5.

Then, we compared the pharyngeal teeth details of PP, SW and PS and quantified the maximum opening distance between their pharyngeal teeth and their puncture ability based on the following principle: for the same pressure and a smaller force area, the greater the pressure. We quantified the following indicators: the maximum opening width between pharyngeal teeth (MOWPT), the development degree of hook pharyngeal teeth (DDHPT) and the grinding surface area of pharyngeal teeth (GSAPH); these values were calculated using the following formulas:

$$ MOWPT= TW/ HW $$

$$ DDHPT= TL^{\prime }/ TL $$

$$ GSAPH=S^{\prime }/S $$

where *TW* represents the maximum width distance between pharyngeal teeth, *HW* represents head width; *T* represents average length of 5 lateral pharyngeal teeth, *TL*′ represents the average length of the hooked portion at the tip of the lateral 5 pharyngeal teeth; and *S* represents the basal area of all pharyngeal teeth, *S*′ represents the grinding surface area of all pharyngeal teeth. Further information on these parameters is provided in Additional Figure 2.

We quantified the foraging-related traits (Additional Table 8, 20 measured traits and 17 standardized traits) of all fishes (*n* = 32) in Section 2.7 to explore whether a correlation exists between these traits, and these indicators included the TNC (the total number of captures), TNI (the total number of ingestions), TNSF (the total number of spitting fish) and SR by Spearman’s correlation in SPSS 21.0.

### Statistical analyses

The mean ± standard deviation (SD) was used to represent the unannotated quantitative data, and the other data are annotated in the table or graph notes. One-way analysis of variance (ANOVA) was used to analyse the data of three independent experiments. Spearman’s correlation method was used to analyse the correlation. All the data obtained above were measured and calculated using SPSS software version 19. Tukey’s test was used to analyse the difference. We conducted principal component analysis (PCA) of the Z-scores of these indicators using IBM SPSS Statistics (version 21.0, Armonk, New York, United States). All graphs were generated by the Origin software version 2019b or SPSS software version 19.

## Results

### Morphology

Regarding the external and skeletal morphology, most PS traits were between PP and SW as supported by Tukey’s test or PCAs (Figs. 1 and 2, Additional Table 3). It is worth mentioning that the tail length of PS is longer than that of the parents, which is the main reason why PCA2 of PS is different from that of the parents in the PCA of external morphology (Additional Tables 3 and 5). Specific morphological descriptions are provided in Additional result 1.

### Comparison of foraging habits

In Fig. 3, for small fish, the FL of PP was highly significantly (*P* < 0.01) higher than those of SW and PS. The latter two ingested very few small fishes, and no significant difference (*P* = 0.161) was found between them. For tough periphytic algae, the FL of SW was highly significantly (*P* < 0.01) higher than those of PP and PS. PP did not ingest periphytic algae, and some PS individuals may have ingested a small amount of periphytic algae; however, no significant difference was observed between them (*P* = 0.082). The FL of periphytic algae (*Spirogyra*) in SW was relatively low, probably because it was not the most suitable periphytic algae for SW; however, the FL of SW on *Spirogyra* algae was still highly significantly higher than that of PP or PS (Figs. 3e and 4e).

Interestingly, we found a large amount of small fish debris in the PS aquarium tank (Fig. 3c), while little debris was noted in the tanks with SW and PP, suggesting that one of the reasons for low intake of PS to small fish was spitting fish.

### Hybrid vs parents in foraging fish or periphytic algae

In the PS vs SW experiment of foraging periphytic algae, the FAT of PS was highly significantly higher (*P* < 0.01) than that of SW (Fig. 4c). The reason why the data presented double peaks may be due to the individual differences in periphytic algae foraging of PS, i.e., either they were interested at the beginning or not interested at all. The AF of PS was significantly lower (*P* = 0.02) than that of SW (Fig. 4d), the FL was highly significantly lower (*P* < 0.01) than that of SW (Fig. 4e), and the FE was significantly lower (*P* = 0.037) than that of SW (Fig. 4f). In summary, PS showed low interest in foraging for periphytic algae and had low foraging efficiency.

In the PS vs PP experiment for foraging fish, the SRFA (*P* = 0.219) and SRTA (*P* = 0.167) of PS were not significantly different from those of PP; the SR of PS was highly significantly higher (*P* < 0.01) than that of PP (Fig. 4g); the FAT (*P* = 0.459) and the FST (*P* = 0.161) of PS were not significantly different from those of PP; the FAT2 was highly significantly higher (*P* < 0.01) than that of PP (Fig. 4h); and the AF of PS was highly significantly lower (*P* < 0.01) than that of PP (Fig. 4i). In summary, PS showed greater interest in first foraging for fish but had a high SR, which caused PS to be negative in later predation.

### Whether the behaviour of hybrid fish spitting fish is persistent

As shown in Fig. 5, at the beginning of the experiment, most PS had the behaviours of catching, spitting and ingesting small fish. However, as the experiment proceeded, the number of PS with these behaviours decreased, and only a few fish retained these persistent behaviours by the end of the experiment (Figs. 5d, e, 6f and 5j); thus, this pattern was the main reason for the decline in the average number of daily captures, spitting and ingestion (Fig. 5g, h and i). In summary, the behaviours of catching, spitting and ingesting small fish by most PS were not persistent.

### Mechanism of fish spitting by hybrid fish

No significant difference (*P* = 0.702) was found between the ANDF of fish meat and small fish in the individuals exhibiting persistent capture behaviours (Fig. 6i). However, the SR of fish meat was significantly lower (*P* < 0.01) than that of small fish (Fig. 6j), suggesting that the spitting behaviour was not caused by bad taste but by chewing difficulty, which may be caused by pharyngeal tooth structure, prey size, and the maximum opening width between pharyngeal teeth. Therefore, the SR of PS to different sizes of meat and fish was quantified. The small fish weighed the same as small-sized meat, and the medium fish weighed the same as medium-sized meat. The results showed that PS did not spit on large, medium and small meat and had a low SR for small fish but a high SR for medium fish (Fig. 6k).

Next, the details of the pharyngeal teeth were compared, and we found that the pharyngeal bone of PP was long and narrow, with widely spaced well-developed conical hooked pharyngeal teeth, and the space was larger between the two pharyngeal bones in the closed mouth. These features are useful for piercing and hooking prey. In contrast, the pharyngeal bone of SW was short and thick, with closely spaced grinding pharyngeal teeth, which were curved and flat at the top, forming a grinding surface, and the space was smaller between the two pharyngeal bones in the closed mouth. These features are useful for grinding periphytic algae. The morphology of the pharyngeal bone in PS was balanced between that of the parents, and it had hooked grinding pharyngeal teeth, which were also intermediate between the parents.

The quantitative results support the above morphological description. The MOWPT of PP was highly significantly higher (*P* < 0.01) than that of SW and PS, and no significant difference was observed between them (*P* = 0.588, Fig. 6l). The DDHPT of PP was highly significantly higher (*P* < 0.01) than that of SW and PS, and PS was highly significantly higher (*P* < 0.01) than SW (Fig. 6m). The GSAPH of SW was highly significantly higher (*P* < 0.01) than that of PP and PS, and PS was highly significantly higher (*P* < 0.01) than PP (Fig. 6n).

Spearman correlation analysis descriptions can be found in Additional result 2. The results of the correlation analysis can be summarized as follows: (1) the larger the PS was, the more fishes it caught. (2) With the increase in capture number, both the ingestion and the spitting increased; the latter increased more, which indicated that more fishes were spitted. (3) The captured fishes can be ingested or spitted, so there was a negative correlation between the ingestion and spitting rate. (4) The spitting rate did not vary with the capture number. (5) The spitting rate of PS was not correlated with the size and shape of its quantified traits.

## Discussion

### Intermediate morphology of hybrid fish

Morphology is often determined by quantitative traits, therefore the morphology of F1 hybrids is general between parents [1, 21]. In this study, PP and SW had disparate feeding habits and foraging traits, and most food habit-related quantitative traits of PS were between parents, but there were also a few superparent traits, such as the longer tail length of PS (Additional Table 3), it may benefit PS’s swimming ability. Interestingly, for PP and SW, the sharp horny front jaw is an invisible trait, which is not exhibited by PS (Fig. 1). In addition, our quantitative analysis screened out a large number of food habit-related traits, which provided a reference for subsequent food habit-related morphological studies of other fishes (Additional Tables 2–7).

### Enhancement of postzygotic ecological isolation of parents with contrasting ecological niches

Hybridization generally occurs between closely related sympatric species, and they generally have ecological niche differentiation and adaptive traits [1, 36, 37], which leads to hybrids with intermediate traits that cannot well adapt to the ecological niche of the parents [10, 21]. In previous studies, the ingestion of parental resources by hybrids or their growth performance in the parental environment was generally the mean of both parents [10, 18, 21, 24], which meant the ecological isolation between them was not that great. However, in the above studies, there were no hybrid cases of contrasting ecological niche parents, such as carnivorous and phytophagous individuals. As described in the introduction, our results support previous predictions, namely, the intermediate foraging morphology of PS did not result in intermediate foraging performance for parental resources, and PS could hardly forage for parental resources. A similar example has been found in natural hybridization of sunfishes, as hybrid individuals exhibited kinematics intermediate between those of the two parental species. However, performance assays indicated that hybrids display performance most similar to that of the worse-performing species for a given parental resource [25]. The difference is that in our research, the poor foraging performance of PS for parental resources was caused not only by the decline in PS foraging ability but, more importantly, by the decrease in foraging activity.

The food habit of a species depends not only on heredity and environment, but also experience [38–43]. PS showed less interest in foraging for periphytic algae from the beginning of the experiment, which may be innate. Interestingly, however, PS showed interest in foraging small fishes at the beginning of the experiment, while after the first successful capture, PS had difficulty ingesting the fish, which led to the subsequent decrease in foraging activity. This result may be experiential.

### Mechanism of hybrid fish spitting fish

The behaviour of PS spitting fish is one of the highlights of this study. Two mechanisms may explain why PS spitted small fish: the small fish tasted bad or were difficult to chew. When fed with bone-free meat, there was almost no spitting behaviour of PS, which invalidated the first hypothesis. Chewing difficulties may be caused by two factors, namely, the prey size is too large or there is a defect in their own traits. No spitting was found when PS was fed different sizes of meat, but when PS was fed medium fish with the same weight as medium meat, they still had a higher spitting rate. However, when PS was fed small fish with the same weight as that of small meat, their spitting rate decreased significantly. In summary, for easy-to-chew meat, regardless of its size, PS will not spit it out; however, for difficult-to-chew fish, PS can only ingest smaller individuals that are easy to chew, indicating that the mechanism of fish spitting in PS may be related to defects in its chewing function.

Therefore, we quantified the foraging-related traits of 32 fishes in Section 2.7 to explore whether a correlation exists between these traits and the spitting rate. Unfortunately, we did not find any correlation between any trait and the spitting rate, indicating that other non-self factors may also affect the spitting rate, such as the size of food. Regrettably, in Section 2.7, we did not realize that we should subdivide the size of the fish food, as subsequent Additional experiments proved that it could indeed affect the spitting rate. Interestingly, in PS, the spitting rate did not vary with the total capture number, indicating that regardless of how strong or weak the PS were in predation, they had a similar and weaker ability to ingest small fish, which also reflected the defects of their chewing function. In summary, the above analysis suggested that the mechanism of fish spitting of PS was not related to its own differential traits, and further comparison with parental traits is needed.

Therefore, the structure of PS and parental pharyngeal teeth was further quantified. The function of the pharyngeal teeth of carnivorous Cyprinidae fishes is to puncture food [44], similar to canine teeth, which is reflected in the PP. In contrast, the function of the pharyngeal teeth of herbivorous Cyprinidae fishes is to grind food [44], similar to cheek teeth, which is reflected in SW. Regarding pharyngeal tooth puncture ability, that of PS was between that of the parents but not as good as that of PP, indicating that PS may not reach the threshold of puncture fish. In addition, regarding the maximum width of pharyngeal teeth, that of PS was close to that of SW but significantly smaller than that of PP, suggesting that PS can only chew smaller prey than PP. In conclusion, the difficulty of ingesting small fish by PS may be due to the intermediate pharyngeal tooth traits, which do not effectively enable puncture of fish. Of course, this may not be the only reason, the chewing strength and the tolerance to fish bones of pharynx may also be important reasons for PS spitting fish, but these indicators are not easy to test.

## Conclusion

This study preliminarily proved our prediction that contrasting ecological niches between parents will lead to great ecological isolation by comparing the foraging level and foraging behaviour of carnivorous PP, herbivorous SW and their hybrid (PS) associated with parent resources. The external morphology and skeletal morphology of PS were between those of the parents, but the intermediate foraging morphology of PS was not associated with intermediate foraging performance for parental resources, and PS could hardly forage for parental resources. The poor foraging performance of PS for parental resources was caused not only by the decline in PS foraging ability but, more importantly, by the decrease in foraging activity. Interestingly, PS has obvious fish-spitting behaviour, which is a typical example of the incompatibility between intermediate traits and genetic behaviors.

## Supplementary Information

**Additional file 4.**

## Data Availability

The datasets used or analysed during the current study are available from the corresponding author on reasonable request.
