# Supplementary material for: Contrasting ecological niches lead to great postzygotic ecological isolation: a case of hybridization between carnivorous and herbivorous cyprinid fishes
Source: Front Zool. 2021 Apr 21;18:18. doi: 10.1186/s12983-021-00401-4 (PMC8059018; doi:10.1186/s12983-021-00401-4)
Supplement: Supplementary file 4 — Additional file 4. [file 12983_2021_401_MOESM4_ESM.docx]

Additional Information: Contrasting ecological niche differences lead to great postzygotic ecological isolation: a case of hybridization between carnivorous and herbivorous Cyprinidae fishes

Haoran Gu^1^, Yuanfu Wang^1^, Haoyu Wang^1^, You He^2^, Sihong Deng^3^, Xingheng He^4^, Yi Wu^4^, Kaiyan Xing^5^, Xue Gao^5^, Xuefu He^1^ and Zhijian Wang^1*^

^1^Key Laboratory of Freshwater Fish Reproduction and Development (Ministry of Education), Key Laboratory of Aquatic Science of Chongqing, School of Life Sciences, Southwest University, Chongqing 400715, China;

^2^Shanghai Synchrotron Radiation Facility, Shanghai Advanced Research Institute, Chinese Academy of Sciences, Shanghai 201204, China

^3^Liangshan Kehua Water Ecology Company Limited, Xichang 615000, China

^4^Sichuan Lubei Biotechnology Company Limited, Chengdu 610011, China

^5^Xichang Agriculture and Rural Affairs Bureau, Xichang 615000, China

*Corresponding author: Zhijian Wang

E-mail address: [Wangzj1969@126.com](mailto:Wangzj1969@126.com)

**Additional** [**method**](javascript:;)**s**

**Additional** [**method**](javascript:;) **1: Comparison of foraging habit**

In small fish feeding experiments, each fish species was divided into 5 parallel groups, and 3 fishes in each parallel group were cultured in a 50 L tank with a water temperature of 20±1°C. The experiment was carried out for 5 days. These fishes fasted for the first two days and were fed with appropriate food for the next two days. On the last day, fishes were fed excessive food, and each parallel group was sampled 2, 4, 6, 8, and 10 hours after feeding. These fishes were then anaesthetized using MS-222 (3-aminobenzoic acid ethyl ester methanesulfonate), and the body weight and chyme weight of each fish were weighed and recorded. In [periphytic](javascript:;) [algae](javascript:;) feeding experiment, we put 15 fishes of each species into a concrete pond (3 m×3 m×0.6 m) filled with tough [periphytic](javascript:;) [algae](javascript:;), and on the fifth day, we caught all fishes [at](javascript:;) [noon](javascript:;) and dissected and weighed them.

**Additional** [**method**](javascript:;) **2: Hybrid vs *P. pingi* in** [**foraging**](javascript:;) **fish**

Each experimental fish was cultured separately in a 50 L tank with a water temperature of 20±1°C and fasted for 2 days. On the third day, we fed 10 small fishes (*S. taeniatus*) to each experimental fish separately; furthermore, to ensure there was an excess amount of food, the experiment ended when 5 small fishes were caught or after 30 min.

**Additional** [**method**](javascript:;) **3: Hybrid vs *S. wangchiachii* in** [**foraging**](javascript:;) **periphytic algae**

These experimental fishes were placed respectively in a 65 L aquarium tank with circulating water at a water temperature of 18±1°C and fasted for 39 h. Next, we placed rocks with periphytic algae in the aquarium tank and collected 9 h of video. Then, we performed anaesthetic dissection and recorded the body weight and chyme weight.

**Additional** [**method**](javascript:;) **4: Whether the behaviour of hybrid fish spitting fish is persistent**

Each PS (n=32) was cultured separately in a 50 L tank with a water temperature of 20±1°C and fasted for 1.5 days. In the afternoon of the second day of fasting, we fed 0.5 g of blood worms to each fish. In the following days, we fed the study fish 10 small fishes (*Carassius auratus*). The small fishes of *S. taeniatus* were used up in the previous experiment, but because it was *C. auratus’s* breeding season, we replaced *S. taeniatus* with *C. auratus*, and PS still had obvious spitting behaviour after catching the small fishes of *C. auratus* provided to each PS at 9 a.m. (small fish were replenished regularly to ensure that they were provided in an excessive amount (more than 5)). After two hours, we counted the number of dead small fish and live small fish and cleaned up all of the small fish. We fed 0.5 g of blood worms to each fish every afternoon to simulate a palatable food shortage in the natural environment but not a complete absence.

**Additional** [**method**](javascript:;) **5: Mechanism explaining why hybrid fish spitted fish**

This was a subsequent Additional experiment, so the size of PS is larger than that before. 30 PS (20.32±4.01 g) was cultured separately in six 50 L tanks (5 PS in each tank) with a water temperature of 19±1°C. In order to eliminate the stress response of PS, they were cultured in the tank for one week before being used for experiment. After fasting for two days, each tank was successively fed with 10 small fishes (*S. taeniatus*, 0.0938±0.0132 g), 10 medium fishes (*Schizothorax davidi*, 0.2628±0.0349 g), 10 small meat (0.1007±0.0136 g, there was no significant difference between this weight and that of small fish), 10 medium meats (0.2432±0.0133 g, there was no significant difference between this weight and that of medium fish), 10 big meats (0.5004±0.0470 g), and the interval between them was 2 h. The above foods were fed one by one to prevent the re-capture of the spit food by different individuals and ending in half an hour. Some groups may catch less than 10 fishes, we chose groups that caught more than 5 fishes for comparison of SR (spitting rate).

**Additional results**

**Additional** **result 1: Specific morphological descriptions**

In external morphology, the head of the PP was relatively slender with a developed branchial membrane, lower lip with higher radian, lower jaw without keratinization, terminal mouth, deeper mouth crack, sparse gill-rakers, bulgier eyeballs and longer snout barbel and maxillary barbel. Comparatively, the head of SW was relatively round with an undeveloped branchial membrane, lower lip with a lesser radian, lower jaw with keratinization, inferior mouth, shallower mouth crack, serried gill-rakers, slightly bulging eyeballs and shorter snout barbel and maxillary barbel. In general, the head traits of PS were between those of PP and SW, but there were some special features. For example, PS had a longer maxillary barbel than both parents and a lower jaw without keratinization, which was the same as PP. In PCA of the external characteristics, PS was between PP and SW (Fig. 1m and Additional Tables 4-5).

In skeletal morphology, the traits of the three fishes were similar to their external morphologies, but there were some special features. For example, PP had a much more developed lower jaw but less developed upper jaw; SW had the opposite traits, and PS was somewhere in between. PP had a well-developed branchiostegal ray, SW had the opposite, and PS was somewhere in between. The relative size of the pharyngeal bone of PP was larger than that of SW, and it was long and narrow, which made the spacing of the pharyngeal teeth distributed above it larger; in contrast, the SW pharyngeal bone was shorter and wider, which made the spacing of the pharyngeal teeth distributed above it tighter, and PS was somewhere in between. In PCA of bone characteristics, PS was between PP and SW (Fig. 3m and Additional Tables6-7).

**Additional result 2:** **Correlation analysis descriptions**

In the correlation analysis of foraging traits and foraging ability of PS (Additional Table 8), we did not find any correlation (P≥0.05) between the standardized traits of PS, and these indicators included the TNC, TNI, TNSF and SR. This result indicated that the shapes of those traits had no correlations with the above 4 indicators. Moreover, we did not find any correlation (P≥0.05) between any measured traits and SR. However, we found that some measured traits were positively correlated (P<0.05) with TNC, TNI and TNSF. Only mouth crack depth (MCD) and GL (gut length) were positively correlated with TNI (P<0.05). For TNC and TNSF, these measured traits, including WL (whole length), HL (head length), SL (snout length), GL (gut length), PHA (articulation axis height of pharyngeal teeth), PHB (distance between external angle and articulation axes of pharyngeal teeth), and PHD (dorsal limb length of pharyngeal teeth), had positive correlations (P<0.05) with both. BW2 (body weight) was positively correlated (P<0.05) with only TNC, and MBL (maxillary-barbel length) was positively correlated (P<0.05) with only TNSF.

In the correlation analysis of the above 4 indicators (Additional Table 9), we found that TNC was extremely significantly positively correlated (P≤0.01) with TNI and TNSF, TNI was extremely significantly positively correlated (P≤0.01) with TNSF, and TNI was extremely significantly (P<0.01) [negative](javascript:;)ly correlated with SR. Thus, we selected these individuals (TNC≥10, n=19) for the same analysis, and the results were similar to those above. However, the TNI and TNSF no longer had significant correlations (P≥0.05), and the TNSF and SR re-established extremely significant positive correlations (P<0.01).

The captured fishes can be ingested or spitted, and TNC, TNI and TNSF had a stronger correlation and the same change trend (Additional Table 9 and Fig. 5), they showed similar correlation with different traits in the correlation analysis of different traits. However, TNC and TNSF had a stronger positive correlation and change trend compared with TNI, and TNI was negatively correlated with SR, so only two traits (MCD and GL) had significant correlation with TNI (Note: These two traits had the strongest correlation with TNC compared with other traits). Mouth crack depth (MCD) is related to mouth size, and they are positively correlated. Gut length (GL) is related to digestion. Neither of these traits was related to the ingestion process and SR of small fishes.

**Additional Figure 1**


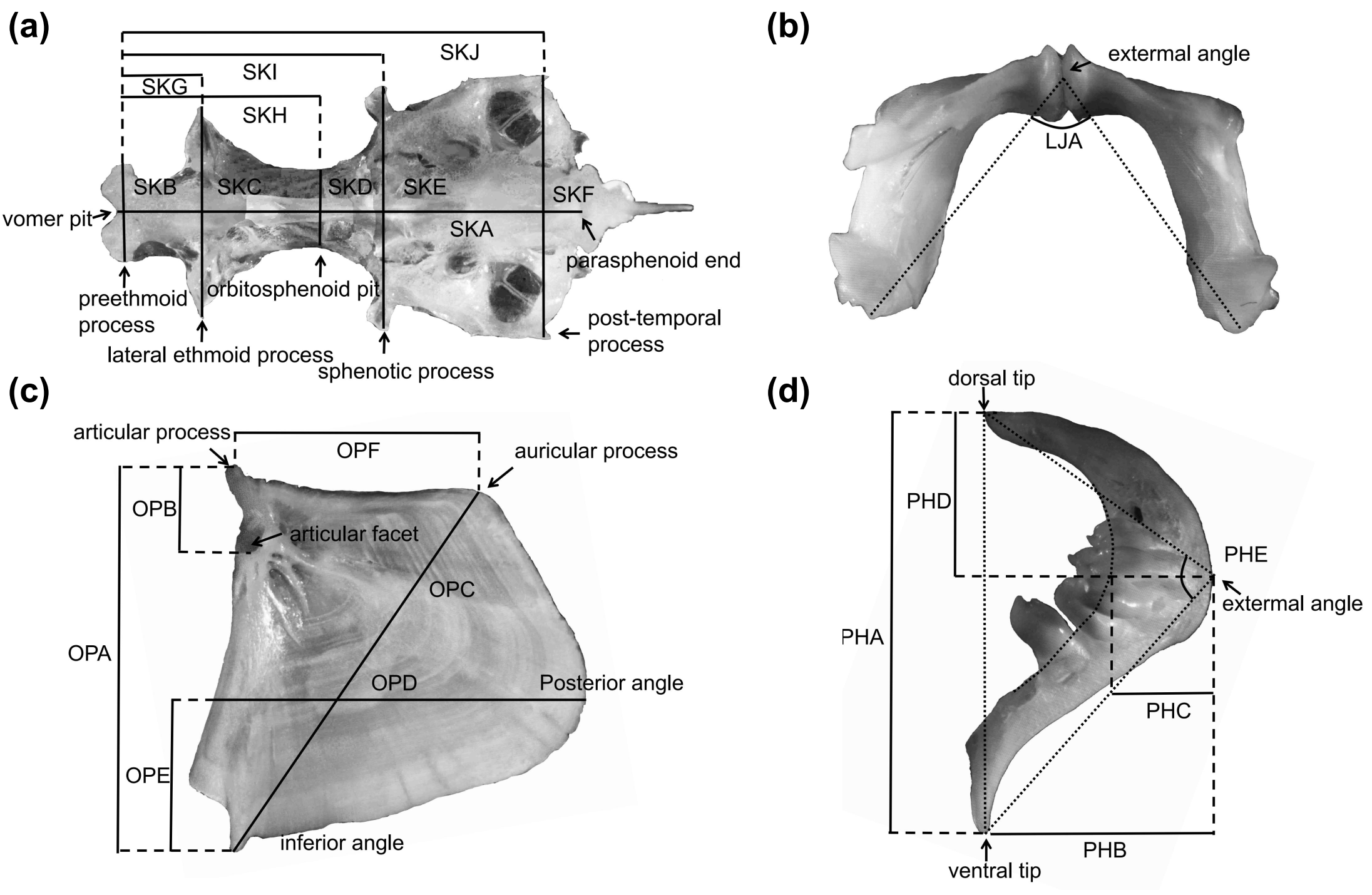


Reference figure for quantifying skeleton. (a) Reference figure for quantifying skull. (b) Reference figure for quantifying dentary. (c) Reference figure for quantifying opercular bone. (d) Reference figure for quantifying pharyngeal bone. These abbreviations are referred to in Additional Table 2.

**Additional Figure 2**


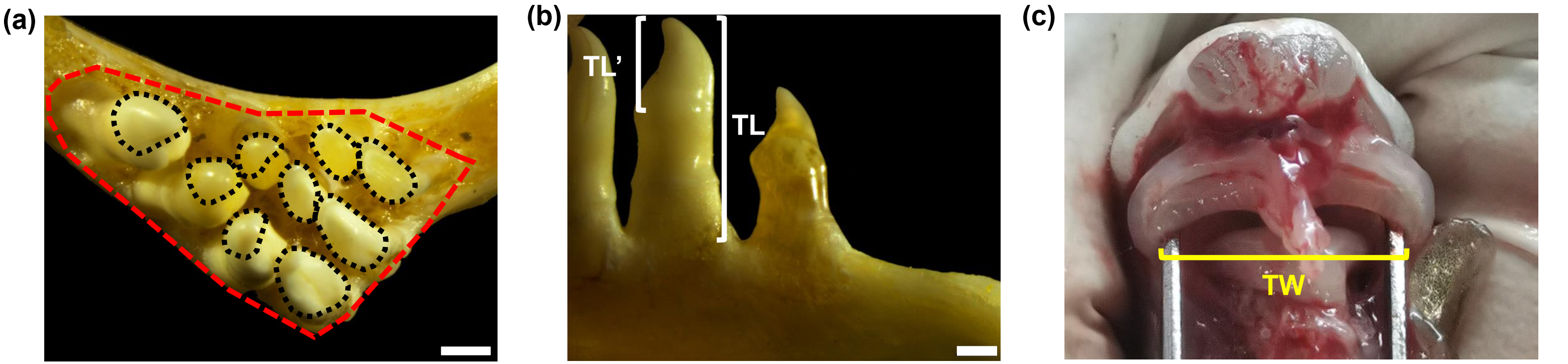


Reference figures for quantifying puncture ability and maximum width distance of pharyngeal teeth. (a) Reference figure for quantifying the grinding surface area of pharyngeal teeth (GSAPH). The area of red curve represents the basal area of all pharyngeal teeth ($S$), and the area of black curve represents the grinding surface area of all pharyngeal teeth ($S'$). (b) Reference figure for quantifying the development degree of hook pharyngeal teeth (DDHPT). TL represents the length of pharyngeal teeth, and TL’ represents the length of hooked portion at the tip of pharyngeal teeth. (c) Reference figure for quantifying the maximum width distance between pharyngeal teeth (TW).

**Additional Table 1** List of abbreviations for all measures and their definitions

| Abbreviation | Items |
| --- | --- |
| FL | Foraging level |
| FAT | First attack time |
| FST | First success time |
| SRFC | The success rate of the first successful capture |
| FAT2 | First attack time after the first successful capture |
| AF | Attack frequency |
| SRTA | The success rate of the total attacks |
| SR | The spitting rate |
| FE | Foraging efficiency |
| ANDF | The average number of daily foraging |
| MOWPT | The maximum opening width between pharyngeal teeth |
| DDHPT | The development degree of hook pharyngeal teeth |
| GSAPH | The grinding surface area of pharyngeal teeth |
| TNC | The total number of captures |
| TNI | The total number of ingestions |
| TNSF | The total number of spitting fish |

**Additional Table 2** List of quantitative characters and their abbreviations

| Items | Abbreviation |
| --- | --- |
| **External characters** | |
| Whole length | WL |
| Body length | BL |
| Body width | BW1 |
| Body height | BH |
| Head length | HL |
| Dorsal fin anterior length | DFAL |
| Caudal peduncle length | CPL |
| Caudal peduncle depth | CPD |
| Snout length | SL |
| Mouth crack depth | MCD |
| Sub-head length (The distance from the posterior end of the gill cover to the anterior end of the lower lip) | SHL |
| Eye diameter | ED |
| Interorbital width | IW |
| Distance of eyeballs | DE |
| Snout-barbel length | SBL |
| Maxillary-barbel length | MBL |
| Number of gillrakers on outer row | GOR |
| Number of gillrakers on inner row | GIR |
| Whether have a sharp horny front jaw ([qualitative](javascript:;) [character](javascript:;), “have”=”1”, “none”=“0”) | SHJ |
| **Skeletal characters** | |
| Opercular bone |  |
| Articular axis height | OPA |
| Length of articular process | OPB |
| Maximum height | OPC |
| Maximum length | OPD |
| Posterior angle height | OPE |
| Distance between superior angle and articulation axis | OPF |
| Pharyngeal bone |  |
| Articulation axis height | PHA |
| Distance between external angle and articulation axes | PHB |
| Maximum width of pharyngeal bone | PHC |
| Dorsal limb length | PHD |
| Angle within the limbs | PHE |
| Dentary |  |
| Angle within the dentary | DEA |
| Skull |  |
| Vomer pit to parasphenoid end | SKA |
| Distance between the two process of preethmoid | SKB |
| Distance between the two process of lateral ethmoid | SKC |
| Distance between the two pit of orbitosphenoid | SKD |
| Distance between the two process of sphenotic | SKE |
| Distance between the two process of post-temporal | SKF |
| Vertical distance between ateral ethmoid process and preethmoid process | SKG |
| Vertical distance between orbitosphenoid process and preethmoid process | SKH |
| Vertical distance between sphenotic process and preethmoid process | SKI |
| Vertical distance between post-temporal and preethmoid process | SKJ |

**Additional Table 3** Standard characters of four fishes

| Items | PP | SW | PP×SW |
| --- | --- | --- | --- |
| WL/BL | 1.1988±0.0126^a^ | 1.2214±0.0137^b^ | 1.2577±0.0157^c^ |
| BW1/BL | 0.1296±0.0068^b^ | 0.1221±0.0014^a^ | 0.1294±0.0055^b^ |
| BH/BL | 0.2096±0.0104^a^ | 0.2221±0.0102^b^ | 0.2081±0.0082^a^ |
| HL/BL | 0.2844±0.0084^c^ | 0.2195±0.0083^a^ | 0.2409±0.0093^b^ |
| DFAL/BL | 0.5410±0.0136^c^ | 0.5081±0.0122^a^ | 0.5083±0.0110^a^ |
| CPL/BL | 0.1494±0.0113^a^ | 0.1633±0.0144^b^ | 0.1514±0.0123^a^ |
| CPD/CPL | 0.6195±0.0525^a^ | 0.6139±0.0632^a^ | 0.6298±0.0618^a^ |
| SL/HL | 0.2459±0.0139^a^ | 0.2732±0.0167^c^ | 0.2629±0.0105^bc^ |
| MCD/HL | 0.2252±0.0174^c^ | 0.0843±0.0066^a^ | 0.1640±0.0147^b^ |
| SHL/HL | 1.0253±0.0101^c^ | 0.8776±0.0296^a^ | 0.9284±0.0198^b^ |
| IW/DE | 0.5155±0.0124^a^ | 0.6778±0.0093^c^ | 0.5863±0.0105^b^ |
| ED/HL | 0.2319±0.0172^a^ | 0.2602±0.0179^b^ | 0.2561±0.0167^b^ |
| IW/HL | 0.2354±0.0091^a^ | 0.3924±0.0207^c^ | 0.3174±0.0210^b^ |
| SBL/HL | 0.2504±0.0149^a^ | 0.1218±0.0208^c^ | 0.2155±0.0183^b^ |
| MBL/HL | 0.2548±0.0189^b^ | 0.1542±0.0211^a^ | 0.2718±0.0234^bc^ |
| NOGR | 17.9667±0.8899^a^ | 25.6000±1.2205^d^ | 20.2000±1.0306^b^ |
| NIGR | 10.6000±0.4983^a^ | 17.8667±1.6526^d^ | 12.7000±1.0554^b^ |
| SHJ | 0 | 1 | 0 |
| OPB/OPA | 0.2067±0.0164^a^ | 0.2378±0.0126^b^ | 0.2349±0.0153^b^ |
| OPC/OPA | 1.1279±0.0381^ab^ | 1.0833±0.0357^a^ | 1.1512±0.0306^b^ |
| OPD/OPA | 1.0072±0.0486^b^ | 0.8057±0.0409^a^ | 0.8418±0.0805^a^ |
| OPE/OPA | 0.4119±0.0638^a^ | 0.4147±0.0226^a^ | 0.5063±0.0513^b^ |
| OPF/OPA | 0.6225±0.0420^b^ | 0.5538±0.0339^a^ | 0.5840±0.0181^ab^ |
| PHB/PHA | 0.3933±0.0244^a^ | 0.4866±0.0141^b^ | 0.4922±0.0214^b^ |
| PHC/PHA | 0.1587±0.0115^a^ | 0.2544±0.0285^b^ | 0.2343±0.0151^b^ |
| PHD/PHA | 0.2462±0.0279^a^ | 0.4519±0.0275^c^ | 0.3611±0.0392^b^ |
| PHE | 92.8319±3.0941^b^ | 91.3234±2.5501^b^ | 87.1146±1.6831^a^ |
| SKB/SKA | 0.1846±0.0172^a^ | 0.2187±0.0087^b^ | 0.1969±0.0148^a^ |
| SKC/SKA | 0.3585±0.0353^a^ | 0.4644±0.0179^c^ | 0.4173±0.0343^b^ |
| SKD/SKA | 0.1270±0.0133^a^ | 0.1954±0.0201^b^ | 0.1663±0.0238^b^ |
| SKE/SKA | 0.5036±0.0276^a^ | 0.5409±0.0246^b^ | 0.5193±0.0179^ab^ |
| SKF/SKA | 0.5259±0.0379^a^ | 0.5702±0.0181^b^ | 0.5428±0.0167^ab^ |
| SKG/SKA | 0.1563±0.0158^a^ | 0.1625±0.0121^a^ | 0.1726±0.0172^a^ |
| SKH/SKA | 0.4098±0.0309^a^ | 0.4074±0.0197^a^ | 0.4229±0.0206^a^ |
| SKI/SKA | 0.6037±0.0462^b^ | 0.5617±0.0163^a^ | 0.5758±0.0096^ab^ |
| SKJ/SKA | 0.9625±0.0409^a^ | 0.9297±0.0132^a^ | 0.9500±0.0177^a^ |
| DEA | 32.3061±4.4547^a^ | 57.463±3.3247^c^ | 42.4467±4.1244^b^ |

The full name of abbreviation should refer to Additional Table 2. Values in the table are represented as Mean ± SD. a,b Values within a row with different superscripts differ significantly at P < 0.01 based on Tukey test. IW/DE is an indicator defined in this study, it can represent the degree of eyeball protrusion, which is related to feeding habits. SHL/HL is an indicator defined in this study, it can represent the location of mouth, which is related to feeding habits. Some of the external trait data we have used in previous study [[1](#_ENREF_1)].

**Additional Table 4** Total variance of the interpretation of external characters

| Component | Initial eigenvalue | | | Sums of squared loadings | | |
| --- | --- | --- | --- | --- | --- | --- |
|  | [sums](javascript:;) | Variance% | Aggregate% | [sums](javascript:;) | Variance% | Aggregate% |
| 1 | 9.049 | 50.273 | 50.273 | 9.049 | 50.273 | 50.273 |
| 2 | 2.275 | 12.64 | 62.913 | 2.275 | 12.64 | 62.913 |
| 3 | 1.327 | 7.374 | 70.288 | 1.327 | 7.374 | 70.288 |
| 4 | 1.188 | 6.601 | 76.888 | 1.188 | 6.601 | 76.888 |
| 5 | 1.016 | 5.645 | 82.533 | 1.016 | 5.645 | 82.533 |
| 6 | 0.739 | 4.106 | 86.639 |  |  |  |
| 7 | 0.576 | 3.198 | 89.837 |  |  |  |
| 8 | 0.539 | 2.994 | 92.831 |  |  |  |
| 9 | 0.339 | 1.884 | 94.715 |  |  |  |
| 10 | 0.269 | 1.497 | 96.211 |  |  |  |
| 11 | 0.16 | 0.891 | 97.102 |  |  |  |
| 12 | 0.125 | 0.695 | 97.797 |  |  |  |
| 13 | 0.102 | 0.568 | 98.365 |  |  |  |
| 14 | 0.082 | 0.455 | 98.82 |  |  |  |
| 15 | 0.071 | 0.394 | 99.213 |  |  |  |
| 16 | 0.053 | 0.292 | 99.505 |  |  |  |
| 17 | 0.046 | 0.254 | 99.759 |  |  |  |
| 18 | 0.043 | 0.241 | 100 |  |  |  |

**Additional Table 5** Component matrix of external characters

| Items | Component | | | | |
| --- | --- | --- | --- | --- | --- |
|  | 1 | 2 | 3 | 4 | 5 |
| Z-score（WL/BL） | 0.206 | 0.818 | -0.015 | 0.272 | -0.209 |
| Z-score（BW/BL） | -0.465 | -0.214 | 0.334 | 0.622 | -0.237 |
| Z-score（BH/BL） | 0.453 | -0.482 | 0.225 | 0.438 | 0.322 |
| Z-score（DFAL/BL） | -0.622 | -0.136 | -0.029 | -0.395 | 0.444 |
| Z-score（HL/BL） | -0.895 | -0.258 | 0.049 | -0.066 | 0.066 |
| Z-score（CPL/BL） | 0.414 | 0.055 | 0.711 | -0.085 | 0.100 |
| Z-score（CPD/CPL） | 0.010 | 0.053 | -0.565 | 0.488 | 0.542 |
| Z-score（SHL/HL） | -0.872 | -0.308 | -0.01 | -0.049 | 0.056 |
| Z-score（SL/HL） | 0.402 | -0.259 | -0.569 | -0.039 | -0.389 |
| Z-score（MCD/HL） | -0.949 | -0.135 | -0.043 | 0.079 | -0.033 |
| Z-score（ED/HL） | 0.357 | 0.451 | 0.077 | 0.150 | 0.300 |
| Z-score（IW/HL） | 0.924 | 0.095 | -0.033 | 0.074 | 0.030 |
| Z-score（IW/DE） | 0.945 | 0.133 | 0.020 | -0.071 | 0.023 |
| Z-score（SBL/HL） | -0.772 | 0.460 | 0.037 | -0.167 | 0.180 |
| Z-score（MBL/HL） | -0.718 | 0.604 | -0.032 | 0.037 | 0.004 |
| Z-score（NIGR） | 0.911 | 0.097 | -0.008 | -0.184 | 0.117 |
| Z-score（NOGR） | 0.938 | 0.058 | -0.022 | -0.137 | 0.11 |
| Z-score（SHJ） | 0.842 | -0.486 | -0.011 | -0.062 | 0.03 |

**Additional Table 6** Total variance of the interpretation of skeletal characters

| Component | Initial eigenvalue | | | Sums of squared loadings | | |
| --- | --- | --- | --- | --- | --- | --- |
|  | [sums](javascript:;) | Variance% | Aggregate% | [sums](javascript:;) | Variance% | Aggregate% |
| 1 | 7.924 | 41.706 | 41.706 | 7.924 | 41.706 | 41.706 |
| 2 | 2.13 | 11.212 | 52.918 | 2.13 | 11.212 | 52.918 |
| 3 | 1.948 | 10.25 | 63.168 | 1.948 | 10.25 | 63.168 |
| 4 | 1.535 | 8.077 | 71.245 | 1.535 | 8.077 | 71.245 |
| 5 | 0.963 | 5.07 | 76.315 |  |  |  |
| 6 | 0.881 | 4.637 | 80.952 |  |  |  |
| 7 | 0.807 | 4.248 | 85.2 |  |  |  |
| 8 | 0.586 | 3.083 | 88.283 |  |  |  |
| 9 | 0.524 | 2.759 | 91.042 |  |  |  |
| 10 | 0.463 | 2.436 | 93.479 |  |  |  |
| 11 | 0.32 | 1.685 | 95.164 |  |  |  |
| 12 | 0.308 | 1.621 | 96.785 |  |  |  |
| 13 | 0.201 | 1.059 | 97.844 |  |  |  |
| 14 | 0.121 | 0.638 | 98.482 |  |  |  |
| 15 | 0.091 | 0.48 | 98.962 |  |  |  |
| 16 | 0.078 | 0.412 | 99.374 |  |  |  |
| 17 | 0.062 | 0.326 | 99.699 |  |  |  |
| 18 | 0.033 | 0.172 | 99.871 |  |  |  |
| 19 | 0.025 | 0.129 | 100 |  |  |  |

**Additional Table 7** Component matrix of skeletal characters

| Item | Component | | | |
| --- | --- | --- | --- | --- |
|  | 1 | 2 | 3 | 4 |
| Z-score (OPD/OPA) | 0.686 | 0.05 | 0.01 | 0.424 |
| Z-score (OPE/OPA) | -0.35 | 0.719 | -0.077 | 0.181 |
| Z-score (OPF/OPA) | -0.847 | -0.105 | 0.234 | -0.2 |
| Z-score (PHB/PHA) | 0.114 | 0.627 | -0.524 | 0.313 |
| Z-score (PHC/PHA) | -0.613 | 0.28 | 0.253 | 0.175 |
| Z-score (PHD/PHA) | 0.826 | 0.306 | -0.193 | -0.212 |
| Z-score (PHE) | 0.894 | 0.117 | -0.098 | 0.028 |
| Z-score (SKB/SKA) | 0.869 | -0.004 | -0.061 | 0.021 |
| Z-score (SKC/SKA) | -0.351 | -0.557 | 0.269 | 0.348 |
| Z-score (SKD/SKA) | 0.71 | 0.128 | 0.303 | 0.294 |
| Z-score (SKE/SKA) | 0.846 | 0.084 | 0.178 | 0.361 |
| Z-score (SKF/SKA) | 0.748 | -0.171 | 0.109 | -0.134 |
| Z-score (SKG/SKA) | 0.535 | 0.018 | 0.639 | 0.18 |
| Z-score (SKH/SKA) | 0.603 | -0.099 | 0.536 | -0.222 |
| Z-score (SKI/SKA) | 0.301 | 0.382 | 0.227 | -0.681 |
| Z-score (SKJ/SKA) | 0.071 | 0.562 | 0.455 | -0.259 |
| Z-score (DEA) | -0.513 | 0.304 | 0.549 | 0.26 |

**Additional Table 8** Correlation analysis of foraging traits and foraging ability of hybrid fish

| Item | TNC | | TNI | | TNSF | | SR | |
| --- | --- | --- | --- | --- | --- | --- | --- | --- |
|  | r | P-value | r | P-value | r | P-value | r | P-value |
| BW2 | **0.364*** | 0.041 | 0.311 | 0.083 | 0.27 | 0.135 | -0.030 | 0.903 |
| BL | 0.262 | 0.148 | 0.183 | 0.315 | 0.168 | 0.357 | 0.056 | 0.820 |
| WL | **0.399*** | 0.024 | 0.274 | 0.129 | **0.355*** | 0.046 | 0.163 | 0.505 |
| BH | 0.332 | 0.063 | 0.270 | 0.134 | 0.252 | 0.164 | -0.02 | 0.935 |
| BW1` | 0.271 | 0.133 | 0.170 | 0.353 | 0.208 | 0.254 | 0.08 | 0.745 |
| HL | **0.395*** | 0.025 | 0.255 | 0.159 | **0.407*** | 0.021 | 0.284 | 0.239 |
| SL | **0.427*** | 0.015 | 0.239 | 0.188 | **0.378*** | 0.033 | 0.178 | 0.466 |
| ED | 0.234 | 0.198 | 0.133 | 0.468 | 0.172 | 0.347 | 0.251 | 0.300 |
| DE | 0.294 | 0.103 | 0.254 | 0.160 | 0.135 | 0.460 | -0.061 | 0.805 |
| IW | 0.292 | 0.105 | 0.216 | 0.236 | 0.148 | 0.418 | 0.031 | 0.901 |
| SBL | 0.109 | 0.551 | -0.05 | 0.786 | 0.218 | 0.230 | 0.327 | 0.172 |
| MBL | 0.309 | 0.085 | 0.183 | 0.317 | **0.366*** | 0.039 | 0.259 | 0.284 |
| MCD | **0.448*** | 0.010 | **0.351*** | 0.049 | 0.33 | 0.065 | -0.025 | 0.918 |
| GW | 0.341 | 0.056 | 0.27 | 0.136 | 0.234 | 0.198 | -0.143 | 0.559 |
| GL | **0.484**** | 0.005 | **0.427*** | 0.015 | **0.372*** | 0.036 | -0.041 | 0.868 |
| PHA | **0.433*** | 0.013 | 0.276 | 0.126 | **0.449**** | 0.010 | 0.359 | 0.131 |
| PHB | **0.445*** | 0.011 | 0.324 | 0.071 | **0.390*** | 0.028 | 0.257 | 0.287 |
| PHC | 0.303 | 0.091 | 0.314 | 0.080 | 0.181 | 0.322 | 0.008 | 0.974 |
| PHD | **0.392*** | 0.027 | 0.171 | 0.349 | **0.488**** | 0.005 | 0.431 | 0.066 |
| PHE | 0.042 | 0.818 | 0.03 | 0.871 | 0.070 | 0.705 | 0.026 | 0.915 |
| WL/BL | 0.185 | 0.311 | 0.118 | 0.522 | 0.233 | 0.200 | 0.173 | 0.479 |
| BH/BL | 0.132 | 0.470 | 0.11 | 0.550 | 0.129 | 0.480 | 0.046 | 0.853 |
| BW1/BL | 0.109 | 0.554 | 0.067 | 0.716 | 0.083 | 0.652 | 0.094 | 0.702 |
| HL/BL | 0.081 | 0.658 | 0.033 | 0.857 | 0.162 | 0.377 | 0.199 | 0.414 |
| SL/HL | 0.213 | 0.242 | 0.07 | 0.703 | 0.161 | 0.378 | -0.012 | 0.96 |
| ED/HL | -0.168 | 0.359 | -0.162 | 0.375 | -0.207 | 0.255 | 0.013 | 0.957 |
| DE/IW | -0.217 | 0.234 | -0.212 | 0.245 | -0.077 | 0.677 | 0.055 | 0.822 |
| IW/HL | 0.137 | 0.454 | 0.107 | 0.561 | -0.031 | 0.866 | -0.153 | 0.533 |
| SBL/HL | -0.048 | 0.796 | -0.141 | 0.440 | 0.095 | 0.605 | 0.234 | 0.334 |
| MBL/HL | 0.146 | 0.426 | 0.076 | 0.680 | 0.213 | 0.241 | 0.082 | 0.737 |
| MCD/HL | 0.07 | 0.702 | 0.06 | 0.745 | 0.014 | 0.938 | -0.175 | 0.475 |
| SHL/HL | -0.091 | 0.620 | -0.14 | 0.445 | -0.017 | 0.927 | 0.217 | 0.373 |
| GL/BL | 0.171 | 0.351 | 0.168 | 0.357 | 0.100 | 0.585 | -0.183 | 0.454 |
| GW/BW2 | 0.295 | 0.101 | 0.244 | 0.178 | 0.248 | 0.171 | -0.025 | 0.92 |
| PHB/PHA | -0.023 | 0.898 | -0.042 | 0.819 | -0.027 | 0.885 | 0.025 | 0.918 |
| PHC/PHA | -0.009 | 0.961 | 0.124 | 0.498 | -0.161 | 0.380 | -0.273 | 0.258 |
| PHD/PHA | 0.104 | 0.570 | -0.028 | 0.878 | 0.217 | 0.233 | 0.146 | 0.552 |

TNC: Total number of captures. TNI: Total number of ingestions. TNSF: Total number of spitting fishes. SR: Spitting rate. GW: Gut weight. GL: Gut length. * indicates P<0.05, significant correlation, ** indicates P<0.01, extremely significant correlation. “r”: correlation coefficient. These abbreviations are referred to in Additional Table 2.

**Additional Table 9** Correlation analysis between TNC, TNI, TNSF and SR of hybrid fish

| Item | TNC | | | TNI | | | TNSF | | | SR | | |
| --- | --- | --- | --- | --- | --- | --- | --- | --- | --- | --- | --- | --- |
|  | r | P-value | | r | P-value | | r | P-value | | r | P-value | |
| TNC | 1.000 | | / |  | |  |  | |  |  | |  |
| TNI | **0.844**** | | 0.000 | 1.000 | | / |  | |  |  | |  |
| TNSF | **0.844**** | | 0.000 | **0.482**** | | 0.005 | 1.000 | | / |  | |  |
| SR | -0.234 | | 0.197 | **-0.637**** | | 0.000 | 0.211 | | 0.246 | 1.000 | | / |
| These individuals (TNC≥10, n=19) participated in the following correlation analysis | | | | | | | | | | | | |
| Item | TNC | | | TNI | | | TNSF | | | SR | | |
| TNC | 1.000 | | / |  | |  |  | |  |  | |  |
| TNI | **0.546*** | | 0.016 | 1.000 | | / |  | |  |  | |  |
| TNSF | **0.740**** | | 0.000 | -0.078 | | 0.752 | 1.000 | | / |  | |  |
| SR | 0.274 | | 0.257 | **-0.601**** | | 0.006 | **0.823**** | | 0.000 | 1.000 | | / |

TNC:Total number of capture. TNI:Total number of ingestion. TNSF:Total number of spitting fish. SR: Spitting rate. * indicates P<0.05, significant correlation, **indicates P<0.01, extremely significant correlation. “r”: correlation coefficient. In calculating the correlation between SR and those traits, we selected only individuals whose TNC was greater than 10 in 9 days to eliminate individuals with few catches but high SR.

**References**

1. Gu HR, Wan YF, Yang Y, Ao Q, Cheng WL, Deng SH, Pu DY, He XF, Jin L, Wang ZJ: **Genetic and morphology analysis among the pentaploid F-1 hybrid fishes (Schizothorax wangchiachii female x Percocypris pingi male) and their parents.** *Animal* 2019, **13:**2755-2764.
